# Quality of life and patient experience in Black women with alopecia

**DOI:** 10.1097/JW9.0000000000000203

**Published:** 2025-05-01

**Authors:** Annyella Douglas, Jayson Suriano, Neda Nikbakht

**Affiliations:** a Department of Dermatology and Cutaneous Biology, Thomas Jefferson University, Philadelphia, Pennsylvania

**Keywords:** alopecia, black, emotions, hair loss, Hairdex survey, women

**What is known about this subject in regard to women and their families?**

- Hair loss is prevalent in Black women, yet how it affects quality of life and patient experience in this population has not been well studied.
- Black women are unlikely to visit the dermatologist when experiencing hair loss.
- There is limited data assessing Black women’s hair loss experience as dermatology patients.

**What is new from this article as messages for women and their families?**

- Black women’s quality of life is negatively affected due to their hair loss.
- Among Black women with hair loss, the largest negative impact is on patient’s emotional experience.
- Black women perceive that they spend increased money to conceal and treat their hair loss with increased spending going toward treatment they feel did not help.

Hair loss is common in Black women, but there is much unknown about how it affects their quality of life (QoL) and patient experience. To investigate this, we performed an institutional review board–approved survey study from June to September 2021. We contacted 420 Black women over age 18, who were diagnosed with alopecia at a single academic center from 2015 to 2020, and conducted a phone survey, resulting in 100 respondents. Patients were surveyed using the modified English version of the Hairdex Questionnaire to determine the impact of alopecia on QoL (Supplementary Table 1, https://links.lww.com/IJWD/A68). This survey is standardized to assess alopecia-related QoL and consists of 5 unique domains: functioning, symptoms, self-confidence, stigmatization, and emotions.^1^ In addition to the Hairdex survey, our study team created 21 additional questions further assessing patient discovery of hair loss, lifestyle adjustments to hair loss, patient–provider relationships, and opinions on treatment outcomes (Supplementary Table 2, https://links.lww.com/IJWD/A69).

Among 100 Black women who responded to the surveys, 28.0% had nonscarring and 72.0% had scarring alopecia subtypes, with the most common diagnosis of central centrifugal centripetal alopecia (35.0%). The mean score of the Hairdex Questionnaire was 29.5, which included emotions (41.8), symptoms (32.9), self-confidence (27), functioning (20.2), and stigmatization (18.3). The emotions domain had the highest normalized mean score compared with other domains and this difference was statistically significant (*P* < .0001)^1,2^ (Fig. 1).

**Fig. 1.**
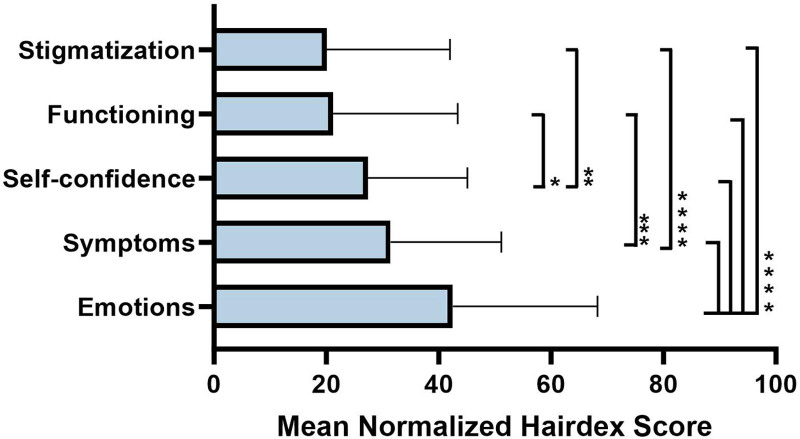
Mean normalized Hairdex scores for each of the 5 domains. The difference between each domain score was evaluated using analysis of variance tests. A higher score is associated with poorer QoL. The highest score was for emotions (****P < .0001 vs all domains), followed by symptoms (***P < .0002 vs functioning and ****P < .0001 vs stigmatization), followed by self-confidence (**P < .004 vs stigmatization and *P < .02 vs functioning), followed by functioning, and stigmatization with the lowest scores. Error bars indicate standard deviation.

The additional questions created by our study team showed that 58.2% of patients perceived increased financial spending to either conceal or treat their alopecia with 55.1% perceived spending a lot of money on treatments that did not help with their hair loss (Fig. 2). To conceal hair loss, 44.9% of patients reported wearing a wig, 45.9% wore a head covering such as a hat or bandana, and 64.3% wore specific styles to conceal their hair loss. On average, 60.4% of patients sought dermatological care more than 3 months after noticing hair loss. Although not statistically significant, the 16 patients who had biopsies were more likely to experience improved symptoms or symptom resolution, be happy with the outcome of their dermatologist’s treatment plan, feel confident that their dermatologist was adequately trained to treat their hair type, and trust their dermatologist’s treatment plan. Despite biopsy, less than half of patients experienced complete resolution of their symptoms or were happy with their treatment outcome (Fig. 2).

**Fig. 2.**
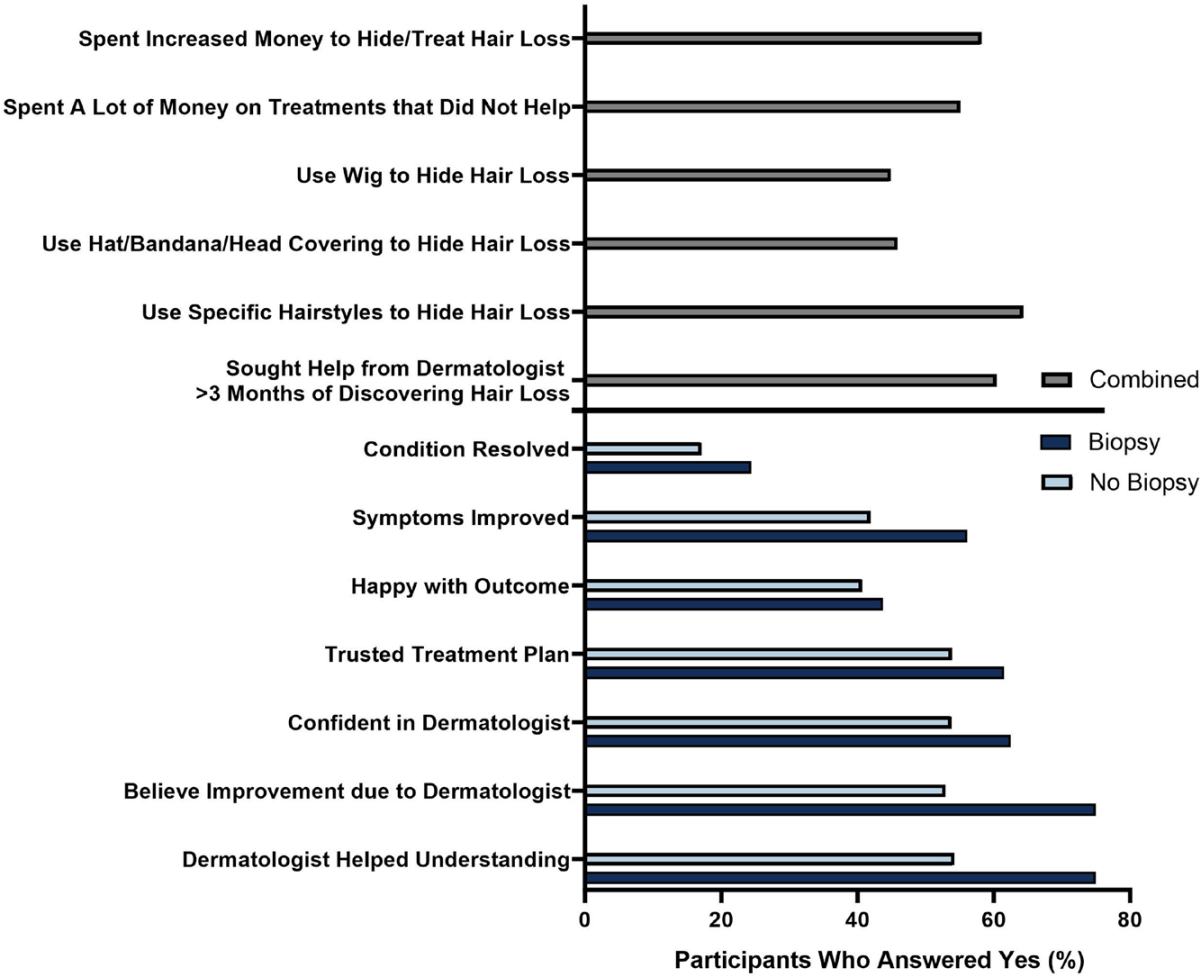
Top: all participants combined (*N* = 100). The percentage of surveyed participants who answered “Yes” to our study team’s questions assessing perception, lifestyle adjustments, and patient–provider relationship for the statements listed on the left. Bottom: response from participants who received a scalp biopsy (*n* = 16) compared with participants who did not (*n* = 84) for the statements listed on the left. Fisher exact test did not reveal statistical significance between the 2 groups.

This study illustrates the strain of alopecia on Black women and their experience in the dermatology office. We find that alopecia has an emotional burden that negatively affects patients’ QoL and potentially increases their financial burden.^3,4^ Black women may feel they are spending considerable amounts on treatments to conceal and address their alopecia, often without seeing any improvement. Scalp biopsies could improve the patient experience and should be considered when managing patients.^5^ Limitations in this study include recall bias, a small sample limited to a single academic center, and a nonvalidated 21-question survey. However, this study underscores areas for improvement in managing Black women with hair loss.

## Conflicts of interest

None.

## Funding

None.

## Study approval

This study was approved by the Institutional review board.

## Author contributions

AD, JS, and NN wrote and prepared the manuscript for publication. JS and AD contributed to data analysis and interpretation for the final manuscript. NN contributed to the conceptualization, editing, and writing of the manuscript.

## Data availability

All data generated or analyzed during this study are included in this article. Further enquiries can be directed to the corresponding author.

## Supplementary data

Supplementary data related to this article can be found at http://links.lww.com/IJWD/A68 and http://links.lww.com/IJWD/A69.

## Supplementary Material


