# Supplementary material for: Quality of life and patient experience in Black women with alopecia
Source: Int J Womens Dermatol. 2025 May 1;11(2):e203. doi: 10.1097/JW9.0000000000000203 (PMC12047871; doi:10.1097/JW9.0000000000000203)
Supplement: Supplementary file 1 [file jw9-11-e203-s001.pdf]

Supplemental Table 1: Hairdex Survey (modified English version)

Hairdex scales	Questions related	
**1.- Stigmatization**	22. My hair loss is a problem for the people I love	B
	26. I feel humiliated by my hair condition	B
	32. I have problems sitting in the bus, cinema or theater where others can see my hair closely	B
	34. I feel like an outsider because of my hair condition	B
	38. Others make fun of me because of my hair condition	B
	42. Others talk behind my back about my hair condition	B
	46. My hair condition is the main feature people notice about me	B
	48. I haven't been taken seriously with my hair condition by my physician	B
**2.- Functioning**	2. The state of my hair has an influence on how well I sleep	B
	4. My hair condition interferes with my work or my leisure activities	B
	5. My hair condition affects my social life	B
	8. I tend to stay at home because of my hair loss	B
	11. My hair condition affects how close I can be with those I love/people close to me	B
	14. I tend to do things alone due to my hair loss	B
	16. Water (bathing, washing) bothers my scalp or my hair	B
	20. My hair condition affects my interactions with others	B
	25. I tend to interact less with others because of my hair condition	B
	29. My hair loss interferes with my sex life	B
	37. My hair condition makes it difficult to achieve as much I usually do	B
**3.- Self-confidence**	31. I can handle my hair condition	A
	33. Despite my hair condition I am content with myself	A
	35. Despite my hair condition life is worth living	A
	39. Overall I have a good self-confidence despite my hair condition	A
	40. Others show understanding for my hair condition	A
	41. I go to the hairdresser/hair-salon as often as usual despite my hair/scalp condition	A
	43. Compared to others I am lucky to have this hair	A
**4.- Symptoms**	1. My scalp hurts	B
	7. My scalp burns or stings	B
	10. My scalp itches	B
	18. My scalp is in bad condition	B
	19. My scalp is irritated	B
	24. My hair is very sensitive	B
	27. My scalp bleeds	B
**5.- Emotions**	3. I worry that my hair loss may be something serious	B
	6. My hair condition makes me feel depressed	B
	9. I worry that the condition of my scalp may lead to scars/disfiguration	B
	12. I am ashamed due to my hair	B
	13. I worry that my hair loss may get worse	B
	15. My hair condition makes me angry	B
	17. My hair condition makes showing affection difficult	B

	21. My hair loss is embarrassing me	B
	23. My hair condition is frustrating me	B
	28. The condition of my scalp/hair makes me annoyed and cranky	B
	30. My hair conditions annoys me	B
	36. I worry that I may look old because of my hair condition	B
	44. My hair condition is disfiguring me	B
	45. I hate my hair when I see it in the sink, in the hairbrush or on the sofa	B
	47. I look in the mirror every morning and evening to see if my hair condition has changed	B

Legend:

The survey utilizes a five-item Likert scale, where the answer options are 0 = never, 1 = seldom, 2 = sometimes, 3 = often and 4 = always to rate each item asked. The items in the self-confidence domain are inverse and must be reversed and recomputed before analyses because a high rating is a positive "answer" or positive impact on QoL as opposed to all other high ratings indicating a negative impact on QoL. Domain scores and total scores are then linearly transformed to represent a 0-100 point scale, where 0 is the lowest possible score and 100 is the maximum possible score.

A.- A high score a POSITIVE "answer" or positive impact in QoL.

B.- A high score a NEGATIVE "answer" or negative impact in QoL.
