# Supplementary material for: Quality of life and patient experience in Black women with alopecia
Source: Int J Womens Dermatol. 2025 May 1;11(2):e203. doi: 10.1097/JW9.0000000000000203 (PMC12047871; doi:10.1097/JW9.0000000000000203)
Supplement: Supplementary file 2 [file jw9-11-e203-s002.pdf]

Supplemental Table 2: Additional questions created by study team to assess patient perceptions, lifestyle adjustments, and patient-provider relationships for Black women with hair loss

**Additional Questions**
1. Who first discovered your hair loss
2. Where did you first seek out help when you noticed hair loss
3. What other resources have you used to assist in treatment of your hair loss
4. How long after experiencing hair loss did you seek out help from a dermatologist
5. I use a wig to hide my hair loss
6. I use a hat/bandana/head covering to hide my hair loss
7. I use specific hairstyles to hide my hair loss
8. I spend an increased amount of money to hide &/or treat my hair loss than I did prior to my hair loss
9. I spent a lot of money on hair care treatments that did not help with my hair loss
10. I spent a lot of money on hair care treatments that I am not sure help with my hair loss
11. I spent a lot of money on hair care treatments that help with my hair loss
12. I have seen hair regrowth after being seen by a dermatologist
13. I have had improvement in symptoms such as itch, burn, tingling after being seen by a dermatologist
14. My condition has gone away after being seen by a dermatologist
15. I am happy with the outcome of my dermatologist's treatment plan
16. I was confident that my dermatologist was properly trained to treat my hair type
17. I trust my dermatologist's treatment plan
18. I wish I had been seen by a dermatologist sooner
19. The dermatologist helped me to understand the cause and treatment options for my hair condition
20. I think the improvement in my condition was not due to a dermatologist
21. I have not seen any improvement in my hair loss

Legend:

Items 1-4 are open-ended free format response questions. Items 5-21 are binary responses with Yes or No answer options.
